# An Effective Oral Nanodelivery Material for Curcumin: Ingenious Utilization of Gastrointestinal Absorption Characteristics

**DOI:** 10.3390/molecules30122536

**Published:** 2025-06-10

**Authors:** Qiuxu An, Yuanyuan Liu, Guodong Liang, Yuewu Wang, Fengying Liang, Yunyang Bai, Chaolu Eerdun, Riqing Cheng, Haifeng Zhang, Xiaojie Lv

**Affiliations:** 1 College of Pharmacy, Inner Mongolia Medical University, Hohhot 010110, China; 17861903533@163.com (Q.A.); 15849820060@163.com (Y.L.);; 2 College of Basic Medicine, Inner Mongolia Medical University, Hohhot 010110, China

**Keywords:** fullerene, intestinal absorption barrier, nanocarrier, charge reversal, cell-penetrating peptides

## Abstract

Curcumin exhibits compromised bioavailability upon oral administration due to its inherent limitations, including low aqueous solubility, poor membrane permeability, and chemical instability. Inspired by the efficient mechanism by which viruses penetrate mucus and cells, we constructed an electrically neutral and hydrophilic nanocarrier (C60-CPP5/Pser@CUR) using fullerene C60 as the matrix modified with cell-penetrating peptides and phosphoserine. CPP5 facilitates efficient cellular internalization of therapeutic agents, while the incorporation of phosphoserine serves as a charge reversal strategy. This design enables dynamic surface charge modulation to enhance curcumin’s trans-barrier delivery efficiency. Systematic in vitro and in vivo evaluations demonstrated that the synthesized carrier significantly improved the synergistic effects of mucus penetration and cellular uptake. The Caco-2 cellular uptake of curcumin-loaded carriers was 2.26 times higher than that of free drugs. In a single-pass intestinal perfusion study in rat models, this nanocarrier significantly enhanced the absorption of curcumin in the duodenal and colonic regions. In the in vivo experiments, compared with free curcumin, its C_max_ and AUC_0–t_ achieved improvements of 2.60 times and 14.70 times, respectively. This virus-mimetic platform dynamically adapts to micro-environmental demands through charge reversal mechanisms, effectively overcoming sequential biological barriers and providing a robust strategy for oral delivery of hydrophobic therapeutics.

## 1. Introduction

Oral administration is not only convenient but also offers good patient compliance; however, after the drug is taken orally and absorbed in the small intestine, it needs to overcome three barriers: the chemical barrier, the physical barrier, and the biological barrier [1,2]. The chemical barriers encompass the harsh gastrointestinal (GI) environment, including variable pH levels and digestive enzymes, while the physical barriers involve the intestinal mucus layer and tightly packed epithelial cells. Additionally, biological barriers arise from intestinal efflux pumps (e.g., P-glycoprotein), drug-metabolizing enzymes, gut microbiota, etc. These multilayered challenges impose stringent demands on a drug’s solubility, stability, and permeability, restricting its clinical translation [3,4,5]. To address these limitations, nanocarriers have emerged as a promising strategy for oral drug delivery. They protect drugs from degradation in the GI tract, enhance mucus penetration and epithelial permeation through optimized size or surface modifications [6], improve drug dispersion and solubility via nanoscale formulation [7], and enable co-delivery of efflux inhibitors to counteract pump-mediated drug expulsion, thereby collectively enhancing bioavailability and therapeutic efficacy.

Researchers revealed that negatively charged hydrophilic particles can rapidly penetrate the mucosal layer, while positively charged nanoparticles show higher cellular uptake efficiency by epithelial cells [8]. However, carriers focusing solely on overcoming a single barrier fail to simultaneously achieve both mucosal penetration and efficient epithelial absorption.

Intriguingly, viruses efficiently traverse both mucosal and epithelial barriers. Further studies attribute this capability to their unique surface architecture: a mixture of positively and negatively charged amino acid groups endows viruses with hydrophilicity and electroneutrality, enabling evasion of mucosal entrapment and clearance [9]. Upon penetrating the mucosal layer, viruses utilize specialized fusion proteins to enhance intestinal epithelial entry.

Inspired by these findings, bioengineered materials imitating viral surface properties have been developed. When loaded with drugs, these materials effectively traverse the dual barriers of the intestinal mucosal layer and epithelial cells, significantly improving oral drug absorption and bio-availability [10,11,12]. Virus-like particles are nanoscale structures that imitate the natural virus structure but lack the viral genome. They exhibit good bio-compatibility and immunogenicity, making them valuable in drug delivery applications [13]. In recent years, researchers have exploited the characteristics of viruses to develop biomimetic nanocarriers for oral drug delivery. Yang et al. [14] designed a surface ligand-switchable, virus-mimicking, multifunctional nanocarrier (Pep/Gal-PNP). Following oral administration, this carrier can expose trans-membrane peptide fragments via a charge reversal mechanism in the acidic gastrointestinal environment, efficiently traversing the intestinal mucosal barrier and targeting liver cells under physiological pH conditions. This design not only enhances the intestinal absorption efficiency of the drug but also achieves precise delivery to specific organs, demonstrating promising application prospects. Wu’s study [15] introduces P-R8-Pho NPs, a biomimetic nanoparticle system that efficiently delivers insulin orally by overcoming mucus and epithelial barriers. The nanoparticles’ unique design allows them to penetrate mucus and then enter cells upon encountering IAP in the intestine, leading to improved insulin absorption and hypoglycemic effects. Zhang et al. [16] prepared and characterized hydrophilic, electrically neutral MSN-NH2@COOH/CPP5 nanoparticles that mimic viruses. The modified nanoparticles exhibited no significant toxicity in preliminary in vitro and in vivo studies. These virus-mimicking nanoparticles can effectively overcome the barriers of the mucus layer and intestinal epithelium for the oral delivery of protein and peptide drugs.

In 1985, H.W. Kroto et al. [17] discovered fullerenes, a class of carbon nanomaterials characterized by a hollow spherical structure, among which C60 (a spherical 32 hedron) exhibits the highest stability, with a large specific surface area and exceptional structural robustness. The molecular structure of fullerenes contains both C–C and C=C, conferring strong reducing properties. Although poorly water-soluble in their native form, fullerenes can be functionalized to yield water-soluble derivatives with enhanced bio-compatibility, making them promising candidates for drug delivery applications [18]. Additionally, their intrinsic antioxidant capacity enables synergistic therapeutic effects when combined with pharmaceuticals [19].

Curcumin (CUR) is a naturally occurring diketone phenolic compound extracted from the rhizome of Curcuma longa (Zingiberaceae family), demonstrating multifaceted pharmacological properties including antitumor, anti-inflammatory, antioxidant, choleretic, and hypolipidemic activities [20]. Despite its broad therapeutic potential, CUR has not yet been approved for clinical applications due to critical pharmacokinetic limitations [21].

Inspired by viral structural strategies to overcome the dual physiological barriers of intestinal absorption, we developed a fullerene-based nanocarrier co-modified with cell-penetrating peptide KLPVM (CPP5) [16] and phosphoserine (Pser). This design achieved electroneutral surface properties and enhanced hydrophilicity, enabling efficient CUR encapsulation while ensuring mucus penetration. Following intestinal alkaline phosphatase (IAP)-triggered Pser cleavage at the epithelial interface, the nanocarrier underwent from neutrality reversal to positive [22,23], simultaneously exposing CPP5 with validated membrane-penetrating capability [15]. This study systematically evaluates the bioavailability enhancement of curcumin through rationally designed nanocarriers and aims to develop a novel delivery platform specifically designed to address the challenges associated with orally administered drugs exhibiting poor membrane permeability, thereby enhancing their gastrointestinal absorption and therapeutic efficacy.

## 2. Results and Conclusions

### 2.1. Characterization of Nanocarriers

#### 2.1.1. Physicochemical Properties

**Figure 1:**
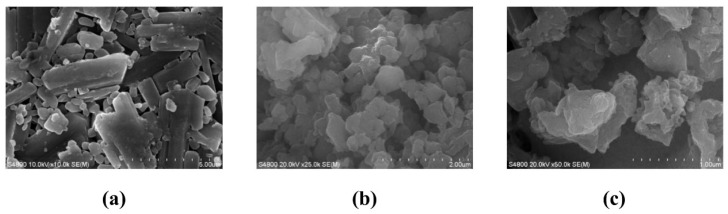
SEM images of (**a**) C60. (**b**) C60-COOH. (**c**) C60-CPP5/Pser.

**Figure 2:**
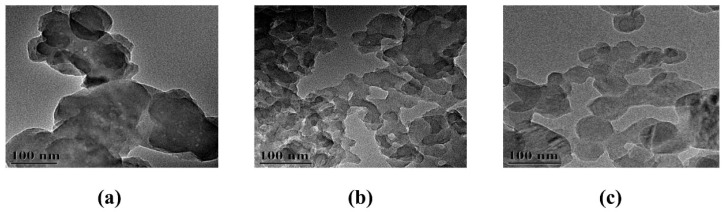
TEM images of (**a**) C60. (**b**) C60-COOH. (**c**) C60-CPP5/Pser.

The nanocarriers presented as loose powders, with C60, C60-COOH, and C60-CPP5/Pser exhibiting black, tan, and yellowish-brown coloration, respectively, accompanied by a sequential reduction in average particle size (Table 1). Due to the nonpolar nature and high specific surface area of pristine C60, its unsaturated surface tends to induce aggregation. Electron microscopy revealed quasi-spherical morphologies across all nanocarriers, with CPP5 and Pser modifications preserving this structural characteristic. The polydispersity index (PDI) values ranged between 0.2 and 0.4, indicating moderately homogeneous yet non-uniform particle distributions. Although surface modifications enhanced hydrophilicity, theoretically mitigating aggregation [24] (Figure 1 and Figure 2), zeta potential effects may facilitate drug-induced agglomeration, resulting in compromised dispersion and impaired drug absorption. This heterogeneity in particle distribution necessitates future optimization through improved synthesis protocols and process design refinement to achieve enhanced uniformity.

**Figure 3:**
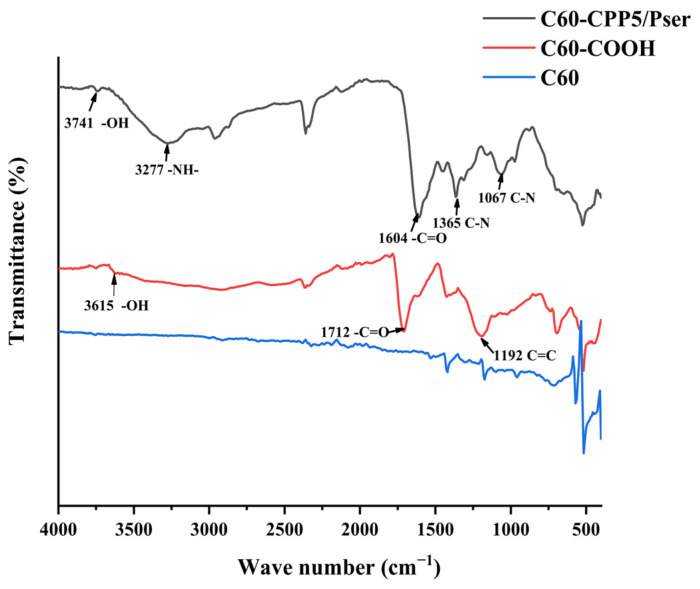
Infrared spectra of C60, C60-COOH, and C60-CPP5/Pser.

In the IR spectrum of C60 (Figure 3), the stretching vibration peak of C=C bonds appears at 1430 cm^−1^, while the absorption bands at 576 cm^−1^ and 527 cm^−1^ correspond to weaker interatomic interactions with larger carbon/carbon distances. Compared to C60, the C60-COOH derivative exhibits a prominent absorption peak at 1712 cm^−1^, characteristic of the carboxyl C=O stretching vibration. For C60-CPP5/Pser, the C=O stretching vibration shifts to 1604 cm^−1^, accompanied by increased absorption intensity and spectral redshift, which is attributed to hydrogen bond association between adjacent molecules. The N-H stretching vibration is at 3277 cm^−1^, and the absorption peaks of the C-N stretching vibration at 1067 cm^−1^ and 1365 cm^−1^ are both characteristic absorption peaks of amide bonds. This phenomenon also obscures the characteristic N-H absorption peak.

**Figure 4:**
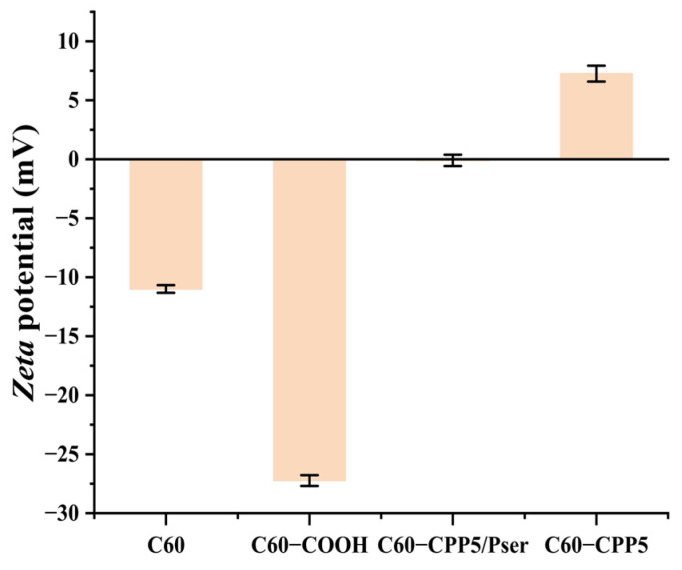
Zeta potential of C60, C60-COOH, C60-CPP5, and C60-CPP5/Pser (*n* = 3).

Zeta potential measurements (Figure 4) reveal distinct surface charge properties: C60 (−11 ± 0.33 mV), C60-COOH (−27.23 ± 0.46 mV), C60-CPP5/Pser (−0.1 ± 0.48 mV), and C60-CPP5 (7.62 ± 0.67 mV). The electroneutrality of C60-CPP5/Pser arises from the mutual cancellation between protonated amino groups on CPP5 and deprotonated phosphate groups on Pser. Comparative analysis of C60-CPP5 and C60-CPP5/Pser confirms the successful surface modification of fullerenes with CPP5 and Pser. Furthermore, based on literature reports [21,22] and our potentiometric data, we propose that charge reversal occurs when surface phosphoserine groups dissociate, leaving CPP5 as the dominant surface component, thereby converting the carrier from electroneutral to positively charged.

#### 2.1.2. Hydrophilicity Test

**Figure 5:**
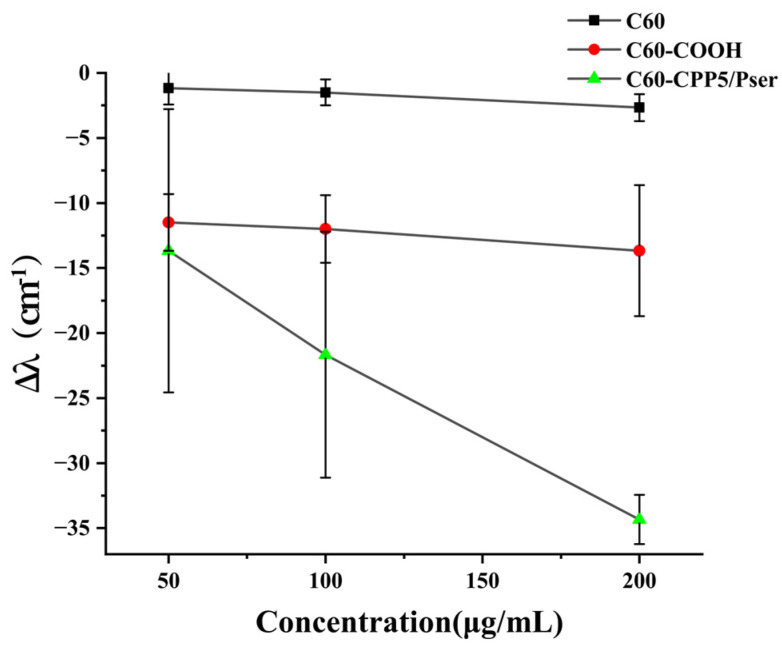
The maximum absorption wavelength of nanocarriers co-incubation with coomassie brilliant blue solution (*n* = 3).

C60 solution exhibited inherent hydrophobicity, which is demonstrated by the absence of blue shift in the maximum absorption wavelength of Coomassie Brilliant Blue (CBB) across varying concentrations. Following carboxyl modification of C60, a concentration-dependent blue shift in the maximum absorption wavelength was observed. Similarly, upon functionalization with CPP5 and Pser, the C60-CPP5/Pser composite also demonstrated a concentration-dependent blue shift in the absorption spectrum (Figure 5). These results indicate that the hydrophilicity of the carrier is enhanced after group modification, among which the C60-CPP5/Pser system shows the most pronounced hydrophilic characteristics.

#### 2.1.3. Antioxidant Activity Assessment

**Figure 6:**
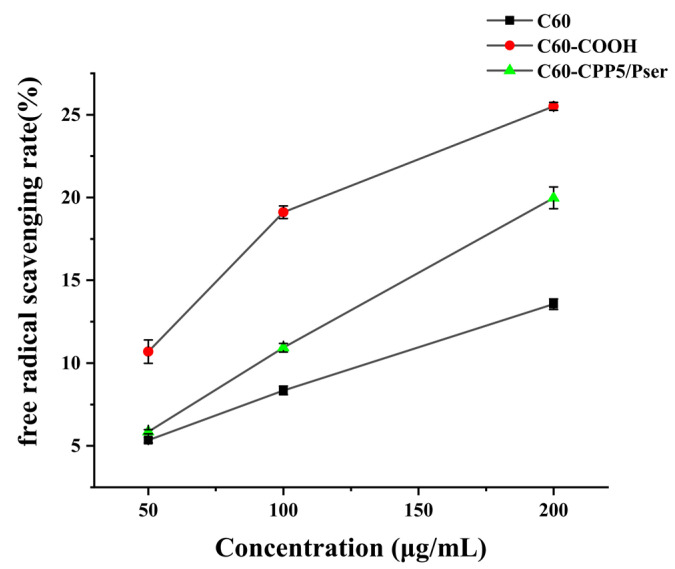
Free radical scavenging curves of nanocarriers (*n* = 3).

With increasing solution concentration, C60, C60-COOH, and C60-CPP5/Pser exhibited a significant enhancement in free radical scavenging activity, among which C60-COOH demonstrated the highest radical scavenging efficiency at 25.51% (Figure 6). It can be preliminarily inferred that the antioxidant properties of C60 were improved after functional group modification.

The observed phenomenon may be attributed to two primary factors. Firstly, unmodified C60 exhibits strong hydrophobicity, leading to aggregation in aqueous or biological media, which shields active sites and reduces radical scavenging efficiency. Carboxyl functionalization enhances hydrophilicity, thereby exposing more reactive surfaces. Secondly, the antioxidant capacity of C60 originates from its unique delocalized π-electron system, which efficiently captures free radicals. The carboxyl group modification elevates electron affinity through its electron-withdrawing effect, intensifying the electron-deficient characteristics of C60. This facilitates the acceptance of unpaired electrons from free radicals. Concurrently, the conjugated system formed between carboxyl groups and C60 establishes novel electron transfer pathways, optimizing electronic properties to accelerate radical scavenging processes. The decrease in antioxidant activity following CPP5/Pser addition is hypothesized to originate from the lower redox potential of the C60-CPP5/Pser complex. In solution-phase systems, the nanoparticles demonstrate a propensity for interparticle aggregation or agglomeration, resulting in the formation of larger particulate assemblies. These aggregated C60-CPP5/Pser configurations may exhibit compromised accessibility to free radicals, thereby attenuating their radical-scavenging capacity and ultimately diminishing antioxidant efficacy [25,26].

### 2.2. Drug Loading and Releasing

Based on orthogonal experimental results, the optimal conditions for preparing C60-CPP5/Pser@CUR via the thin-film dispersion method were determined as a CUR to C60-CPP5/Pser feeding ratio of 1:15 (mole-to-mole ratio), ultrasonication time of 120 min, and ethanol-to-water solvent ratio of 10:5 (*v*/*v*), achieving an encapsulation efficiency of 87.01% and a drug loading capacity of 5.8%.

**Figure 7:**
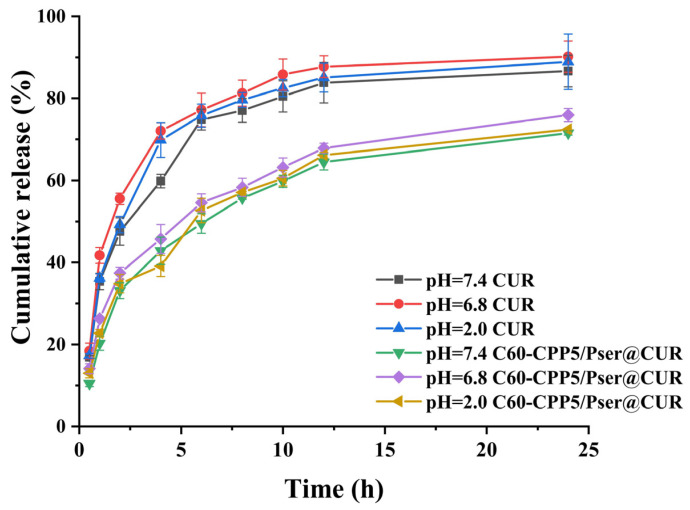
In vitro release of CUR (*n* = 3).

In the drug release experiment, the CUR exhibited rapid release, with nearly 80% cumulative release within 4 h. At 24 h, the cumulative release rates in pH 7.4, 6.8, and 2.0 media reached 86.64%, 90.17%, and 88.89%, respectively. In contrast, the CUR release from C60-CPP5/Pser@CUR was significantly slower, showing only 40% release at 4 h and 24 h cumulative release rates of 71.56%, 75.92%, and 72.37% under pH 7.4, 6.8, and 2.0 conditions, respectively (Figure 7). The in vitro release data demonstrated optimal fitting to the Weibull distribution function across all pH conditions (Table 2). The β parameter < 1 in the Weibull model is interpreted as evidence of a parabolic release profile for CUR (characterized by initial rapid release followed by gradual deceleration), consistent with Fickian diffusion mechanisms, which fundamentally reflects the dynamic equilibrium between carrier/drug interfacial interactions and time-dependent release kinetics. Notably, although 0.5 h rapid release occurred from the nanocarrier surface, the cumulative release never exceeded 40%, confirming the absence of burst release effects for C60-CPP5/Pser@CUR in vitro. The delayed release kinetics compared to free CUR originated from π-π stacking interactions between drug molecules and the fullerene surface, where stronger π-π interactions correlated with slower release rates, demonstrating a sustained-release effect. Analysis of T_50_ and T_d_ parameters (Table 3) revealed superior drug release performance at pH 6.8, likely attributable to pH-dependent alterations in the ionization state of fullerene-modified groups. These changes could modulate carrier charge distribution/hydrophilicity or weaken CUR-fullerene binding through partial CUR ionization under this condition.

### 2.3. Cytotoxicity

**Figure 8:**
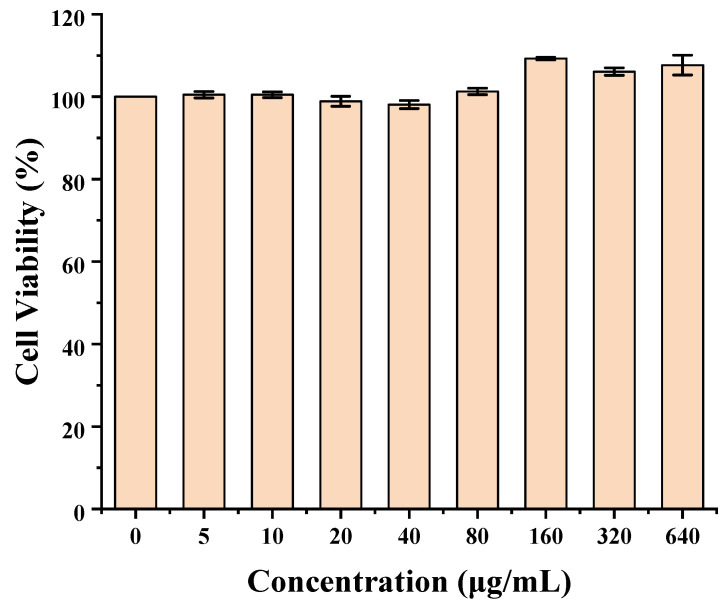
Cytotoxicity of C60-CPP5/Pser (*n* = 6).

To assess the biosafety of C60 as an oral drug delivery carrier, the cytotoxicity of synthesized C60-based carriers toward Caco-2 cells was evaluated with the CCK-8 assay. As shown in Figure 8, the C60-CPP5/Pser nanocarriers exhibited no significant toxicity within the concentration range of 5~640 μg·mL^−1^. Cell viability remained consistently high across all tested concentrations. It initially showed no significant toxicity to this cell, but further verification by other cell lines and animal experiments is still needed. However, this concentration-independent cytotoxicity profile further supports their potential suitability for biomedical applications.

### 2.4. In Vitro Absorption

#### 2.4.1. Cellular Uptake

Single-factor investigation revealed that the uptake of CUR exhibited a time-dependent increase, approximating a linear relationship. Moreover, the cellular uptake by Caco-2 cells showed a concentration-dependent enhancement, with statistically significant differences observed across varying concentrations. Optimal uptake efficiency was achieved at a CUR concentration of 200 μg·mL^−1^. Based on these findings, the experimental parameters for cellular uptake studies were standardized to an incubation time of 20 min and a CUR concentration of 200 μg·mL^−1^.

Under the specified conditions, the cellular uptake of CUR and C60-CPP5/Pser@CUR in Caco-2 cells was quantified as (43.82 ± 1.74) μg·mg^−1^ and (99.11 ± 1.71) μg·mg^−1^, respectively (Table 4). The nanocarrier C60-CPP5/Pser enhanced the cellular uptake of CUR by 2.26 times compared to CUR, demonstrating its significant role in promoting CUR internalization in Caco-2 cells.

#### 2.4.2. Mucus Penetration

**Figure 9:**
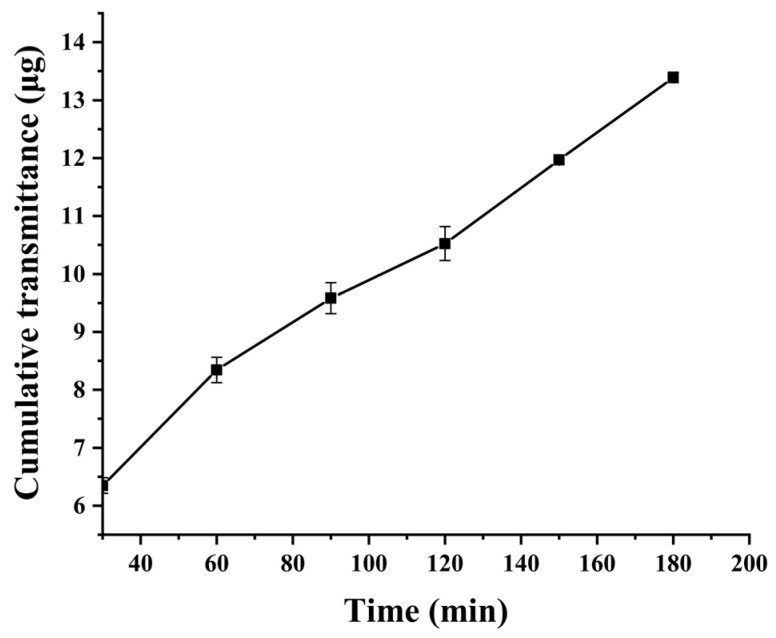
The cumulative transmittance of CUR in C60-CPP5/Pser (*n* = 3).

The intestinal mucus layer, serving as a critical barrier to oral absorption, constitutes a hydrated viscoelastic medium characterized by a mucin fiber network. This reticular architecture mediates the entrapment and clearance of foreign particulates, particularly hydrophobic substances. CUR exhibited negligible mucus penetration capacity, and its cumulative permeation into the receptor chamber remained undetectable. In contrast, the nanocarrier demonstrated significant enhancement in cumulative CUR permeation. As shown in Figure 9, the C60-CPP5/Pser@CUR achieved a cumulative permeation of (13.4 ± 0.1) μg. This enhancement is attributed to the hydrophilic surface properties and near-neutral zeta potential of nanocarriers, which collectively facilitate mucus traversal by minimizing adhesive interactions with mucin fibers.

### 2.5. In Vivo Absorption Effects

#### 2.5.1. Single-Pass Intestinal Perfusion Study

The single-pass intestinal perfusion study in rats demonstrated that after 2 h incubation of CUR solutions with duodenal, jejunal, ileal, and colonic segments, the residual CUR percentages were 99.63 ± 0.34%, 95.63 ± 0.47%, 94.41 ± 1.57%, and 92.90 ± 0.96%, respectively. These results indicated that the intestinal walls exhibited varying degrees of absorption capacity for CUR, among which the colon had the strongest absorption ability, while the duodenum showed virtually no absorption of CUR.

**Figure 10:**
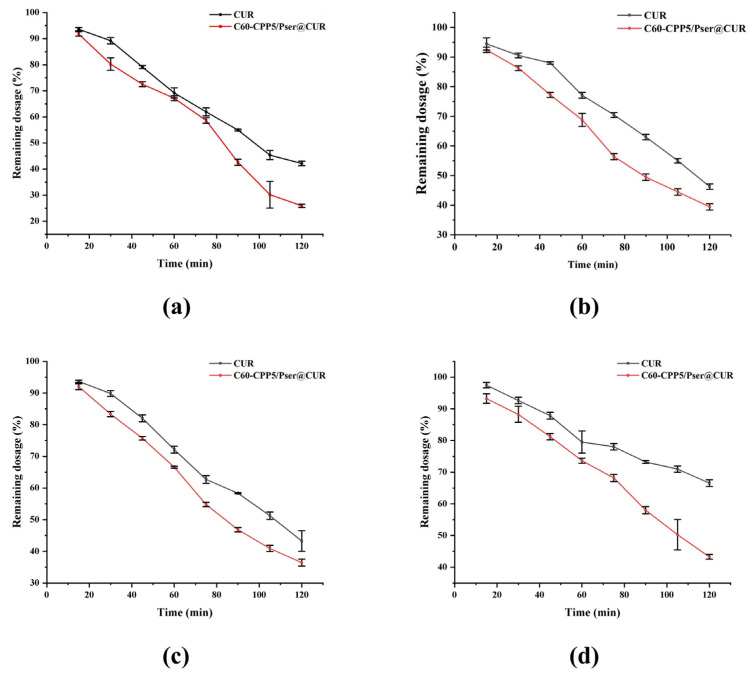
The residual dose of C60-CPP5/Pser@CUR and CUR solution in (**a**) Duodenum. (**b**) Ileum. (**c**) Jejunum. (**d**) Colon of rats at different time periods (*n* = 3).

**Figure 11:**
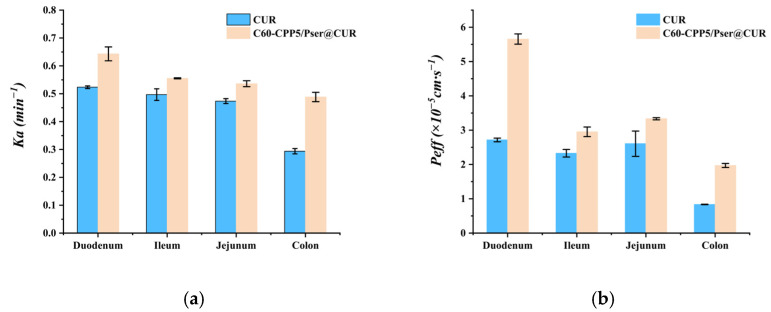
Difference between (**a**) *Ka* and (**b**)Peff of C60-CPP5/Pser@CUR and CUR solution (*n* = 3).

The in situ single-pass intestinal perfusion study in rats demonstrated that the carrier significantly enhanced CUR absorption across all intestinal segments, with absorption kinetics conforming to first-order models. Notably, duodenal and colonic absorption exhibited the most pronounced improvements. At 2 h, the cumulative absorption percentage of the carrier formulation was 1.28 times higher than the CUR solution in the duodenum, followed by 1.12 times in the jejunum, 1.15 times in the ileum, and 1.69 times in the colon (Table 5). The absorption ranking across intestinal segments was duodenum > ileum > jejunum > colon. Comparative analysis of the residual drug amount and absorption rate constant (Ka) revealed that the carrier exhibited the most pronounced enhancement in CUR absorption at the colonic site (Figure 10 and Figure 11). Mechanistically, the carrier’s hydrophilic properties counteracted CUR’s hydrophobicity, while its electrical neutrality minimized mucoadhesive clearance by intestinal mucus. Additionally, surface-conjugated CPPs facilitated transmembrane uptake, and segmental absorption variations were hypothesized to correlate with gastrointestinal alkaline phosphatase distribution patterns.

The effective permeability coefficient (Peff) of C60-CPP5/Pser@CUR in the duodenum, jejunum, ileum, and colon demonstrated 2.08, 1.26, 1.27, and 2.37 time enhancements compared to free CUR solution, respectively. In the assessment of intestinal absorption using the in situ single-pass intestinal perfusion model in rats, compounds with Peff > 2 × 10^−5^ cm·s^−1^ are classified as highly permeable drugs with complete intestinal absorption, whereas those with Peff < 0.3 × 10^−5^ cm·s^−1^ exhibit poor absorption and are categorized as low-permeability agents. These data indicate that C60-CPP5/Pser@CUR qualifies as a highly permeable formulation in specific intestinal segments [27]. Analysis of CUR absorption across intestinal regions revealed superior absorption efficiency in the duodenum, jejunum, and ileum compared to the colon, suggesting regional variability in absorption performance.

#### 2.5.2. In Vivo Pharmacokinetic Study in Rats

**Figure 12:**
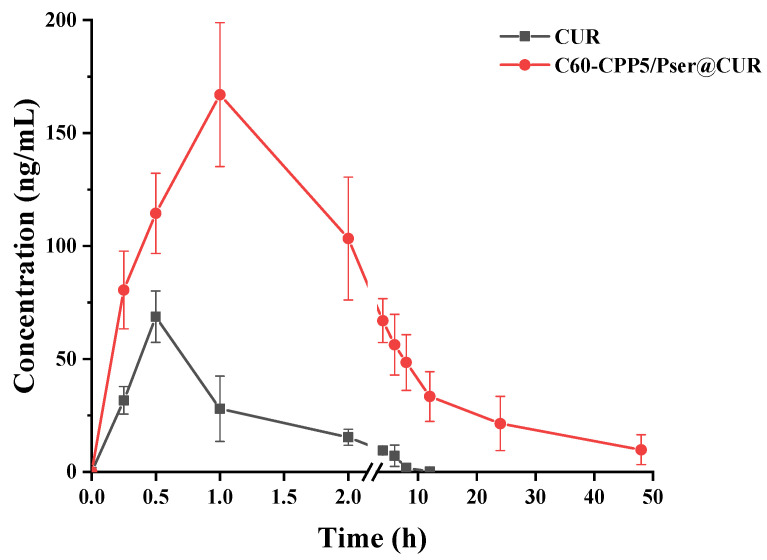
Drug/time curve of C60-CPP5/Pser@CUR and CUR solution in blood.

CUR exhibits extremely low oral bioavailability due to its instability and rapid degradation in vivo, with plasma concentrations barely reaching 1% of the administered dose [28,29]. As shown in Figure 12, blood concentration/time profiles revealed significantly enhanced CUR detection in rats receiving the C60-CPP5/Pser@CUR nanoparticle formulation compared to free CUR. The nanoparticle group demonstrated sustained drug release characteristics, maintaining detectable plasma levels for 24 h, whereas CUR was almost completely eliminated within 4 h. Pharmacokinetic analysis with DAS 2.0 software showed that the C_max_, AUC_0-t_, and MRT_0-t_ of the nanoformulation were 2.60 times, 14.70 times, and 5.95 times higher than those of CUR, respectively. These findings demonstrate that the C60-CPP5/Pser carrier substantially improves CUR bioavailability through dual mechanisms: (1) Protective encapsulation against gastrointestinal degradation and (2) prolonged mucosal adhesion mediated by anti-dilution capacity and sustained-release properties, which collectively reduce intestinal clearance and enhance systemic absorption.

## 3. Materials and Methods

### 3.1. Materials

Fullerene C60(C60) was procured from Aladdin Biochemical Technology Co., Ltd. Shanghai, China, O-phospho-L-serine (Pser), N-Hydroxy succinimide (NHS), 1-(3-Dimethylaminopropyl)-3-ethylcarbodiimide hydrochloride (EDC), and Diethyl bromomalonate were obtained from Yien Chemical Technology Co., Ltd. Shanghai, China, Coomassie Brilliant Blue G-250 (CBB), Curcumin, and Resveratrol were provided by Haohong Biomedical Technology Co., Ltd. Shanghai, China; 1-diphenyl-2-picrylhydrazyl (DPPH·) was provided by Thierry Chemical Industry Development Co., Ltd. Shanghai, China, Cell-penetrating pentapeptides (CPP5, KLPVM peptide) was chemically synthesized by Chu Peptide Biotechnology Co., Ltd. Beijing, China.

### 3.2. Synthesis of the Carrier

The carboxylated fullerene derivative (C60-COOH) was synthesized according to Semenov’s method [30]. Specifically, 10 mg of C60 and 10 mg ethyl bromomalonate were dissolved in 5 mL toluene. After adding 20 molar equivalents of NaH, the solution was evacuated, heated to 60 °C under an inert gas (Ar) atmosphere, and stirred for 3 h. The reaction was terminated with 0.1 mL methanol, resulting in immediate solid precipitation. The product was isolated by vacuum filtration, sequentially washed twice with toluene, 2 M H_2_SO_4_, and deionized water, then vacuum-dried to obtain C60-COOH.

**Figure 13:**
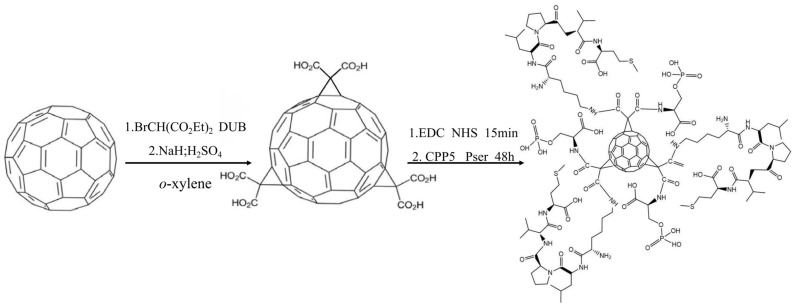
The synthesis route of nanocarriers.

The C60-CPP5/Pser conjugate was prepared through carbodiimide-mediated coupling: 10 mg of C60-COOH was dispersed in 10 mL PBS (pH 7.4), followed by the addition of EDC and NHS at a molar ratio of 1:5:5 (C60-COOH:EDC:NHS). After 15 min activation, a three-fold molar excess of CPP5 and Pser relative to C60-COOH was introduced. The reaction proceeded under light-protected conditions with continuous stirring for 48 h, after which the product was isolated by freeze-drying. The synthetic route is depicted in Figure 13.

### 3.3. Characterization of the Carrier

#### 3.3.1. Physicochemical Properties of the Carrier

The dried sample powder was mounted on an SEM stub, uniformly dispersed, and subjected to high-vacuum evacuation. Prior to imaging, the specimen was sputter-coated with a gold layer to enhance conductivity. Secondary electron micrographs were acquired by Hitachi S-4800 Field Emission Scanning Electron Microscope (Hitachi, Tokyo, Japan).

The sample was dispersed in ethanol at a 1:500 mass ratio and homogenized via ultrasonication. The resulting colloidal suspension was transferred to a quartz cuvette for simultaneous determination of hydrodynamic diameter and zeta potential by SZ-100V Fully Automated Laser Particle Size Analyzer (HORIBA, Ltd., Kyoto, Japan).

The sample suspension was deposited on a copper TEM grid and air-dried to ensure complete ethanol evaporation. Bright-field TEM imaging was performed using a FEI G2-200KV Field Emission Transmission Electron Microscope (Thermo Fisher Scientific, Waltham, MA, USA).

The sample was vacuum-dried with potassium bromide (KBr) powder, homogenized at a 1:100 mass ratio, and pressed into transparent pellets. Fourier-transform infrared (FTIR) spectra were acquired in the 400~4000 cm^−1^ range by IRAffinity-1 Fourier Transform Infrared Spectrophotometer (Shimadzu Corporation, Kyoto, Japan).

#### 3.3.2. Hydrophilicity Characterization

The hydrophilicity of nanocarrier surfaces is correlated with oral absorption efficacy [31]. To evaluate the micro-environmental hydrophilicity of nanocarriers, a co-incubation assay with Coomassie Brilliant Blue G-250 (CBB G-250) dye was performed. Briefly, C60, C60-COOH, and C60-CPP5/Pser were weighed and dispersed in ultrapure water to prepare suspensions at concentrations of 50 μg·mL^−1^, 100 μg·mL^−1^, and 200 μg·mL^−1^, respectively. These suspensions were mixed with an equal volume of 0.1 mM CBB G-250 solution and incubated for 30 min. The maximum absorption wavelength within the 400~800 nm range was measured using UQS-2110007 Double-Beam UV-Vis Spectrophotometer (Mapada Instruments Co., Ltd., Shanghai, China). The shift distance of the maximum absorption wavelength (Δλ) was calculated by comparing the results with a control group containing free CBB solution.

#### 3.3.3. Antioxidant Characterization

The antioxidant capacity can be evaluated through electron transfer mechanisms where antioxidants donate an electron to pair with the lone electron of the DPPH radical (DPPH·), inducing a chromatic shift from purple to yellow at 517 nm. The scavenging efficacy correlates inversely with absorbance intensity, as stronger radical-neutralizing capacity results in greater absorbance reduction [32]. Given C60’s intrinsic electron-accepting properties and well-documented antioxidant activity through high reactivity toward free radicals [33], we assessed the antioxidant performance of C60-CPP5/Pser to determine how structural modifications influence fullerene’s radical-scavenging functionality.

A precise amount of C60, C60-COOH, and C60-CPP5/Pser was accurately individually weighed and dispersed in ultrapure water to prepare nanoparticle suspensions with concentrations of 50 μg·mL^−1^, 100 μg·mL^−1^, and 200 μg·mL^−1^. These suspensions were then mixed with an equal volume of 0.1 mM DPPH· ethanol solution and incubated for 30 min. UV absorption at 517 nm was measured to evaluate the antioxidant activity.

### 3.4. Drug Loading and Releasing

#### 3.4.1. Drug Loading

Analytical measurements were conducted using an Ultimate 3000 high-performance liquid chromatography (HPLC) system (Thermo Fisher Scientific, Waltham, MA, USA). A HPLC method for quantifying CUR was established under optimized chromatographic conditions: a Hypersil GOLD^TM^ C18 column (200 mm × 4.6 mm, 5 μm) was employed with a mobile phase comprising acetonitrile and 0.1% phosphoric acid aqueous solution (48:52, *v*/*v*). The separation protocol operated at a flow rate of 1 mL·min^−1^, column temperature of 25 °C, and detection wavelength of 430 nm. Sample injections (10 μL) were performed using an autosampler, ensuring retention time reproducibility across triplicate runs.

Dried C60-CPP5/Pser was weighed and dispersed in deionized water and absolute ethanol via ultrasonication. Curcumin (CUR) was separately dissolved in ethanol, with the curcumin-to-carrier (mole-to-mole ratio) feeding ratio of 1:15, the ultrasonic time of 120 min, and the ethanol-to-water solvent ratio of 10:5. The CUR solution was then combined with the C60-CPP5/Pser suspension, followed by further ultrasonication. The mixture was subjected to rotary evaporation to form a thin film, which was subsequently reconstituted with 20 mL of deionized water under ultrasonication. The resulting suspension was centrifuged, and the supernatant was collected and lyophilized to obtain C60-CPP5/Pser@CUR. The drug loading methodology was optimized through orthogonal experimental design, with the experimental results analyzed with IBM SPSS Statistics 25.

The drug loading (DL) and entrapment efficiency (EE) were calculated according to Equations (1) and (2) based on the drug content determined using the established CUR method.

(1) $$\;DL\left(\%\right)=\;\frac{The\;amount\;of\;drug\;in\;the\;C60}{Total\;amount\;of\;medication}\;\times 100\%,$$

(2) $$\;EE\left(\%\right)=\frac{Amount\;of\;drug\;encapsulated\;in\;C60}{Total\;amount\;of\;drug\;encapsulated\;versus\;unencapsulated\;in\;C60}\times 100\%,$$

#### 3.4.2. In Vitro Drug Release

CUR and C60-CPP5/Pser@CUR were weighed, respectively, and dispersed in PBS buffer solution, then packed into dialysis bags (MD38.1 kDa). The release media were designed to simulate physiological conditions of the human gastrointestinal tract: pH 2.0 (simulated gastric fluid), pH 6.8 (simulated intestinal fluid), and pH 7.4 (to mimic the alkaline colonic terminus). Release experiments were conducted in PBS buffers (pH 2.0, 6.8, and 7.4) containing 1% Tween-80 and 10% ethanol, with continuous agitation at 120 rpm and 37 °C. Aliquots (0.5 mL) were collected at predetermined intervals (0.5, 1, 2, 4, 6, 8, 12, and 24 h), with the release medium replenished immediately after each sampling to maintain sink conditions. The experiment was repeated three times.

The drug release was quantified using a validated HPLC method. Cumulative release percentages were plotted against time, and release kinetics were analyzed using four models: zero-order kinetics, first-order kinetics, Higuchi equation, and Weibull distribution function. Key parameters, including T_50_ (time required for 50% drug dissolution) and T_d_ (time required for 63.2% drug dissolution), were calculated to evaluate pH-dependent release profiles.

### 3.5. Cytotoxicity

The cytotoxicity of C60-CPP5/Pser was evaluated by the Cell Counting Kit-8 (CCK-8) assay on Caco-2 cells. Briefly, Caco-2 cells were seeded in 96-well plates at a density of 1 × 10^5^ cells·mL^−1^ (100 μL per well) and cultured for 24 h. After removing the medium, various concentrations of C60-CPP5/Pser were added to the wells (six replicates per concentration) and incubated for an additional 24 h. Subsequently, the supernatant was aspirated, and the cell monolayer was gently washed with D-PBS (pH 7.4), then supplemented with 10 μL of CCK-8 reagent and incubated for 3.5 h. The optical density (OD) at 450 nm was measured by a fully automated microplate reader. Cell viability was calculated with Formula (3):

(3) $$\;Cellviability\left(\%\right)=\frac{A_{\;experimental\;group}-A_{\;blank\;group}}{{A\;}_{negative\;control\;group}-{A\;}_{blank\;group}}\times 100\%,$$

(*A_experimental group_* represents OD values of cells treated with varying concentrations of the formulation, *A_blank group_* represents OD values of cell-free wells, *A_negative control group_* represents OD values of wells containing culture medium alone).

### 3.6. In Vitro Absorption

#### 3.6.1. Cellular Uptake

A method for HPLC quantification of CUR in cellular uptake studies was established, which involved validation of specificity, precision, stability, and recovery rates, along with the preparation of a calibration curve (see Supplementary Materials). Cellular samples containing CUR demonstrated excellent linearity within the concentration range of 1~100 μg·mL^−1^, with a regression equation of *y* = 0.972*x* + 0.022 (R² = 0.9995).

Caco-2 cells were seeded into the donor chamber of Transwell inserts at a density of 1 × 10^5^ cell·mL^−1^ and cultured in complete medium. Transepithelial electrical resistance (TEER) was measured on the 0th, 3rd, 5th, 7th, 10th, and 15th days to monitor monolayer integrity. A TEER value exceeding 700 Ω·cm² was established as the threshold for successful formation of the human intestinal epithelial barrier model. Following model validation, the cell surface was equilibrated with PBS for 30 min and rinsed to remove debris. CUR solutions (50, 100, and 200 μg·mL^−1^) were prepared in PBS containing 0.1% DMSO. Subsequently, 500 μL of each CUR solution was added to the apical compartment and incubated for 10, 20, or 60 min. Cellular uptake was terminated by rapid washing with ice-cold PBS to remove surface-bound drug and nonviable cells. Cells were harvested using a cell scraper, lysed, and divided into two aliquots: one for quantifying CUR content via HPLC and the other for determining total cellular protein concentration using a BCA protein assay kit. Cellular uptake was calculated according to Equation (4). Single-factor analysis of uptake time and drug concentration was performed to optimize parameters for subsequent experiments.

Cellular uptake (μg/mg) = *C*_CUR_/*C*_protein_ , (4)

(*C*_CUR_: Intracellular CUR Concentration, *C*_protein_: Intracellular Protein Concentration)

PBS solution containing 200 μg·mL^−1^ CUR (with 0.1% DMSO) was prepared. The established Caco-2 cell monolayer model imitating human intestinal epithelium was equilibrated in pH 7.4 PBS at 37 °C within a CO_2_ incubator for 30 min. After removal of PBS, the cell surface was rinsed three times. Subsequently, 500 μL of CUR solution or nanocarrier dispersion was added to the AP side of the Transwell plate, followed by 20 min of incubation. The uptake process was terminated by ice-cold PBS, and residual solutions were removed by washing. Cells were then scraped into centrifuge tubes, sonicated, and quantified for uptake measurements.

#### 3.6.2. Mucus Penetration Assay

HPLC methodology was developed and validated for the determination of CUR in intestinal mucus. The method demonstrated good linearity within the concentration range of 1~20 μg·mL^−1^, with a calibration curve of *y* = 0.5981*x* − 0.0933 (*R²* = 0.9998).

For mucus preparation, intestinal segments from the distal duodenum to the proximal cecum were excised from rats. Luminal contents were flushed with ice-cold PBS (pH 7.4), and the mucosal layer was mechanically scraped. The harvested mucus was homogenized, centrifuged, and subjected to ethanol precipitation for impurity removal. The resulting pellet was dried and reconstituted in 50 mM sodium carbonate buffer (pH 7.4). After centrifugation at 5000 r·min^−1^ for 15 min at 4 °C, supernatants were collected and pooled for subsequent analysis.

The mucus was uniformly coated into the donor compartment of the Transwell chamber, while PBS (pH 7.4) was added to the receiver compartment. After equilibration at 37 °C for 15 min, 100 μL of 20 mg·L^−1^ CUR suspension and C60-CPP5/Pser@CUR dispersion (containing equivalent drug content) were separately introduced into the donor compartment. The Transwell system was then incubated at 37 °C at 100 rpm for 3 h. At predetermined time intervals, samples were collected and centrifuged, and the CUR concentration was quantified by HPLC. The cumulative mucus permeated amount (*Q*, μg·cm^−2^) and apparent permeability coefficient (*P*_app_, cm·s^−1^) were calculated using Equations (5) and (6):

(5) $$Q=C_{tn}+\sum_{i-1}^{n-1}{0.1C}_{t(n-1)}$$

($C_{tn}$ represents the drug concentration in the receiver compartment at time t; *C*_t(n−1)_ represents the drug concentration in the receiver compartment at time t − 1)

*P*_app_ = (d*Q*/d*T*)/(A × *C*_0_) (6)

(dQ/dT is the cumulative permeation amount per unit time; A is the effective area of the chamber membrane; *C_0_* is the initial concentration of CUR in the donor compartment)

### 3.7. In Vivo Absorption Studies

#### 3.7.1. Single-Pass Intestinal Perfusion

The experimental animals were purchased from SPF Biotechnology Co., Ltd. (Beijing, China, Animal Production License No. SCXK (Jing) 2024-0001) and housed at the Animal Experiment Center of Inner Mongolia Medical University. All animal procedures were conducted in accordance with the guidelines approved by the Ethics Committee of Inner Mongolia Medical University (approval number: YKD202402228).

The methodology for HPLC quantification of CUR in single-pass intestinal perfusion experiments was established to ensure analytical accuracy and reproducibility, then six Sprague–Dawley (SD) rats (body weight: 220 ± 20 g; equal numbers of males and females) were fasted for 24 h with free access to water prior to the experiment. The rats were anesthetized and secured in a supine position on a surgical platform maintained at 37 °C. After abdominal hair removal and disinfection with 75% ethanol, a midline laparotomy was performed to expose the target intestinal segment. A 10 cm intestinal segment was isolated, and incisions were made at both ends. Silicone tubes were inserted into the incisions and ligated. The intestinal lumen was flushed with 0.9% NaCl solution for 5 min to remove luminal contents, followed by equilibration with blank Krebs–Henseleit solution for 15 min (flow rate, 1.5 mL·min^−1^). Subsequently, the test sample was perfused through the intestinal loop. Then, 1 mL intestinal circulation samples were collected at predetermined time points, with an equivalent volume of Krebs–Henseleit solution replenished after each sampling. The collected samples were centrifuged, and the supernatant was mixed with methanol to precipitate proteins. After a second centrifugation, the resulting supernatant was analyzed by HPLC.

#### 3.7.2. Pharmacokinetics Study in Rats

A HPLC-MS method for determining CUR in rat plasma was established with Resveratrol as the internal standard. The analysis was performed on a Q Exactive™ hybrid quadrupole-Orbitrap™ mass spectrometer (Thermo Fisher Scientific, USA) with the following parameters: Chromatographic separation was achieved by Agilent ZORBAX SB-C18 column (150 mm × 4.6 mm, 5 μm) with a mobile phase of acetonitrile and 0.1% formic acid aqueous solution (48:52, *v*/*v*) at a flow rate of 1 mL·min^−1^ while maintaining the column temperature at 30 °C. The mass spectrometer operated in positive ion mode with an electrospray ionization (ESI) source, employing multiple reaction monitoring (MRM) for detection. Key ionization parameters included a capillary voltage of 3.0 kV and nitrogen as the collision gas.

Twelve SD rats (220 ± 20 g, equal numbers of males and females) were fasted for 24 h with free access to water and randomly allocated into two groups (*n* = 6 per group). Both groups received CUR at a dose of 50 mg·kg^−1^ via oral gavage, administered as either a CUR suspension or C60-CPP5/Pser@CUR dispersion. Blood samples (~0.5 mL) were collected from the jugular vein into anticoagulant tubes at 0, 0.25, 0.5, 1, 2, 4, 6, 8, 12, and 24 h after administration. After centrifugation to collect the supernatant, samples were spiked with 20 μL of a 100 μg·mL^−1^ internal standard solution, extracted twice with ethyl acetate for protein precipitation, and the combined organic phases were evaporated using a refrigerated vacuum concentrator. The residue was reconstituted in 100 μL methanol through vortex mixing (4 min) and centrifugation prior to HPLC-MS analysis to quantify the CUR concentration.

### 3.8. Statistical Analysis

The experimental data were expressed as mean ± sd. Comparisons were performed with one-way analysis of variance (ANOVA) with SPSS 25.0 statistical software (IBM Corp., Armonk, NY, USA), and statistical significance was defined as *p* < 0.05.

## 4. Conclusions

Inspired by the viral structural features of viruses to overcome the dual barriers of intestinal mucus and epithelial membranes for oral drug delivery, we developed a charge-reversal nanocarrier (C60-CPP5/Pser). This system takes advantage of its unique surface properties: the zwitterionic coating helps to rapid mucus penetration, while subsequent hydrolysis by mucosal alkaline phosphatase triggers charge reversal to a cationic state. The exposed CPP5 further enhances transmembrane transport via caveolae-mediated endocytosis. These mechanisms were validated by in vitro and in vivo experiments with CUR as a model drug, demonstrating the nanocarrier’s potential for small-molecule delivery. Additionally, the intrinsic antioxidant properties of the fullerene core, which synergistically contribute to therapeutic efficacy, highlight its expanding applications in biomedicine. The therapeutic strategy capitalizes on the inherent antioxidant properties of C60 coupled with enhanced membrane penetration afforded by the delivery system, aiming to develop an oral formulation for intestinal disorders. Our study has yielded encouraging results, demonstrating efficient gastrointestinal delivery of curcumin. However, as illustrated by the data, the carrier exhibits limited regional absorption specificity along the intestinal tract. Future investigations will focus on achieving site-specific release (e.g., colon or inflammatory loci) through targeted modifications of the nanocarrier or fabrication of enteric-coated microcapsules.

While nanocarriers have achieved notable progress in drug delivery, significant gaps remain in terms of successful clinical translation. Comprehensive investigations are required to elucidate the structure/physiological activity relationships of nanocarrier systems, address discrepancies between animal models and the human gastrointestinal tract environment, and evaluate potential side effects arising from their interactions with biological systems. Additionally, the stringent precision demanded in nanopharmaceutical manufacturing poses challenges in maintaining consistency across large-scale production batches. Overcoming these barriers will be critical for realizing the clinical potential of nanocarriers in therapeutic applications. Future research can combine the QbD concept with interdisciplinary technologies (such as AI-driven process optimization) and refer to the successful experience of liposomes/micelles to accelerate their clinical transformation.

## Figures and Tables

**Table 1:** Particle size and PDI of nanocarriers (*n* = 3).

Nanocarriers	Average Particle Size (nm)	PDI
C60	308.4 ± 6.6	0.35
C60-COOH	272.1 ± 2.3	0.39
C60-CPP5/Pser	147.9 ± 5.9	0.42

**Table 2:** In vitro release and equation fitting results under different pH conditions.

pH	Zero-Order Release	*R^2^*	First-Order Release	*R^2^*	Higuchi Equation	*R^2^*	Weibull Equation	*R^2^*
7.4	M_t_ = 0.0397t + 27.4065	0.7297	M_t_ = 66.6058 × (1 − e^−0.01t^)	0.9671	M_t_ = 1.9133 × 1/2 + 9.1879	0.9130	M_t_ = 80.7066 × (1 − e^(−(0.003×(x−1.9257E−8))^0.5697^)	0.9974
6.8	M_t_ = 0.0394t + 31.5089	0.7414	M_t_ = 68.11 × (1 − e^−0.01t^)	0.9351	M_t_ = 1.8878 × 1/2 + 13.6196	0.9185	M_t_ = 96.4292 × (1 − e^(−(0.002×(x−2.1233E−8))^0.4509^)	0.9969
2.0	M_t_ = 0.0393t + 28.8010	0.7367	M_t_ = 69.48 × (1 − e^−0.01t^)	0.9452	M_t_ = 1.8839 × 1/2 + 10.9382	0.9141	M_t_ = 80.76 × (1 − e^(−(0.003×(x−9.1743E−9))^0.6042^)	0.9875

**Table 3:** T_50_ and T_d_ of CUR in carriers under different pH conditions.

pH	T_50_ (min)	T_d_ (min)
7.4	372.87	687.59
6.8	311.79	603.87
2.0	336.43	655.43

**Table 4:** Caco-2 cell uptake of C60-CPP5/Pser@CUR and CUR solution (*n* = 3).

Nanocarriers	Drug Intake (μg/mg)			$\bar{x}$
60-CPP5/Pser@CUR	99.14	97.54	100.97	99.11 ± 1.71
CUR	42.03	44.28	45.46	43.82 ± 1.74

**Table 5:** The difference of Cmax and AUC between CUR and C60-CPP5/Pser@CUR in rats (*n* = 6).

Parameter	C60-CPP5/Pser@CUR	CUR
*t_1/2_* (h)	15.10 ± 2.34	3.97 ± 1.48
*T_max_* (h)	0.5 (0.5~1.0)	1.0 (0.5~1.0)
*C_max_* (μg/mL)	168.20 ± 30.90	64.88 ± 12.02
*AUC_0-t_* [*(*ng/mL)·h]	1555.71 ± 583.42	106.22 ± 21.76
*AUC_0-∞_* [*(*ng/mL)·h]	1698.83 ± 569.61	144.09 ± 25.40
*MRT_0-t_* (h)	13.99 ± 3.23	2.35 ± 0.67
*MRT_0-∞_* (h)	19.27 ± 3.09	5.13 ± 1.36

## Data Availability

The data presented in this study are available upon request from the corresponding authors.

## Supplementary Materials

The following supporting information can be downloaded at: https://www.mdpi.com/article/10.3390/molecules30122536/s1, Figure S1: Specificity test of CUR in release studies; Figure S2: Standard calibration curve of CUR in release studies; Figure S3: Specificity of CUR mucus permeable samples; Figure S4: Standard calibration curve of CUR in mucus samples; Figure S5: Standard calibration curve of CUR in cellular uptake samples; Figure S6: HPLC demonstrating specificity of CUR in single-pass intestinal perfusion samples; Figure S7: Standard calibration curve of CUR in intestinal perfusate samples; Figure S8: Standard calibration curve of phenol red in Krebs-Ringer buffer; Figure S9: Standard calibration curve of CUR in plasma. Table S1: Intraday precision of CUR solution in release studies; Table S2: Interday precision of CUR solution in release studies; Table S3: Stability of CUR solution in release studies; Table S4: Spike recovery of curcumin in release studies; Table S5: Intraday precision of CUR solution for intestinal mucus penetration study; Table S6: Interday precision of CUR solution for intestinal mucus penetration study; Table S7: Stability of CUR solution for intestinal mucus penetration study; Table S8: Recovery of curcumin in mucus matrix for intestinal mucus penetration study; Table S9: Intraday precision of CUR in intestinal perfusate; Table S10: Interday precision of CUR in intestinal perfusate; Table S11: Stability of CUR in intestinal perfusate at room temperature; Table S12. Intraday precision of CUR in plasma; Table S13: Interday precision of CUR in plasma; Table S14: Stability of CUR in plasma at room temperature. In Supplementary S2: Table S1: Percentage of residual dose of CUR in different groups; Table S2: Absorption constant and surface permeability coefficient of C60-CPP5/Pser@CUR in different intestinal segment.
