# Supplementary material for: An Effective Oral Nanodelivery Material for Curcumin: Ingenious Utilization of Gastrointestinal Absorption Characteristics
Source: Molecules. 2025 Jun 10;30(12):2536. doi: 10.3390/molecules30122536 (PMC12196007; doi:10.3390/molecules30122536)
Supplement: Supplementary file 1 [file molecules-30-02536-s001.zip › supplementary materials-S1.pdf]

Methodological Validation results

1. Method for Determining Curcumin Content in Release Studies

1.1 Chromatographic Conditions

Column: HypersilGOLD^{TM} C18 (200 mm × 4.6 mm, 5 μm);

Mobile phase: acetonitrile-0.1% phosphoric acid aqueous solution (48:52, v/v);

Flow rate: 1 mL·min^{-1};

Column temperature: 25 °C;

Detection wavelength: 430 nm;

Injection volume: 10 μL.

1.2 Specificity

C60-CPP5/Pser and CUR were ultrasonically dispersed in anhydrous ethanol, then filtered through a 0.45 μm membrane filter and analyzed by HPLC.

The high performance liquid chromatogram of curcumin is shown as by Figure S1, the C60-CPP5/Pser components did not interfere with the determination of the drug and the curcumin peaks in the curcumin samples were well shaped with no interfering peaks observed in the retention time.

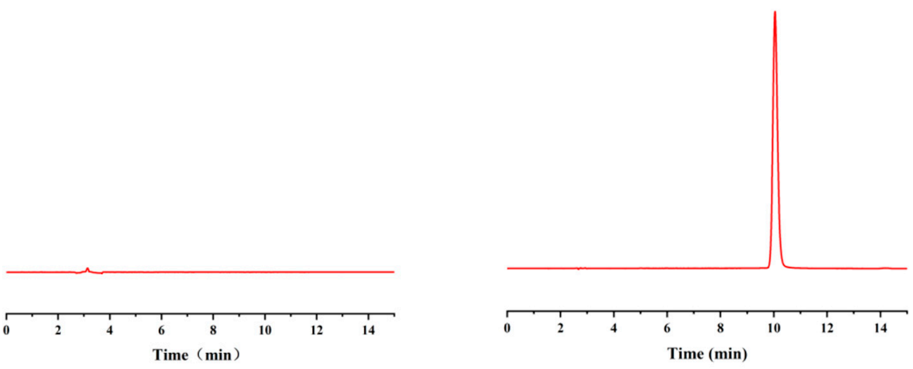

A: Blank solution

B: CUR solution.

Figure S1. Specificity test of CUR in release studies.

1.3 Standard Curve

An accurately weighed quantity of CUR standard was dissolved in ethanol to prepare a 500 μg·mL^{-1} mother liquor. The solution was serially diluted, filtered through a 0.45 μm microporous membrane filter, and analyzed by HPLC. A standard calibration curve was constructed by plotting the peak area of curcumin (*A, mAU*) on the y-axis against its concentration (*C, μg·mL^{-1}*) on the x-axis using linear least-squares regression.

Curcumin in ethanol exhibited good linearity over the concentration range of 1~100 μg·mL^{-1}. The regression equation was:$y = 1.2984x + 0.2601$ ($R^2 = 0.9999$).The standard curve is shown in Figure S2.

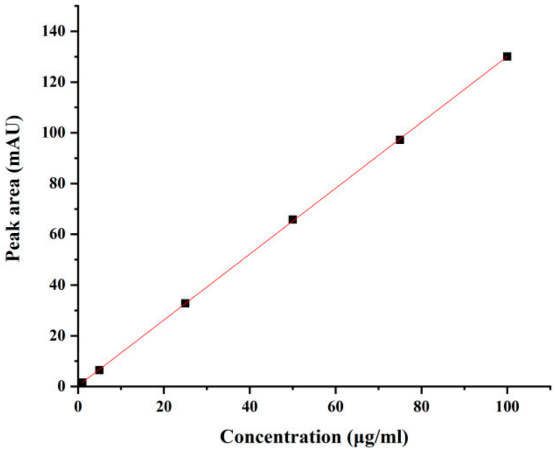

Figure S2. Standard calibration curve of CUR in release studies.

1.4 Precision and Stability

The CUR mother liquor (500 μg·mL^{-1}) was diluted to prepare low, medium, and high concentrations (5 μg·mL^{-1}、50 μg·mL^{-1}、100 μg·mL^{-1}). Each concentration was sampled at 2h intervals and analyzed three times consecutively by HPLC. The RSD was calculated to evaluate intraday precision. The same samples were analyzed once daily over five consecutive days by HPLC, and RSD values were used to assess interday precision.

To evaluate sample stability, a 50 μg·mL^{-1} CUR standard solution was stored at room temperature. Samples were collected at 0, 4, 8, 12, and 20 hours, and CUR content was determined by HPLC. RSD was calculated to assess stability.

The results showed that the intraday RSDs for CUR were 0.91%, 0.58%, and 0.53% for low, medium, and high concentrations, respectively. The interday RSDs were 1.68%, 1.20%, and 1.32%, respectively, indicating good method precision (see Tables 1 and 2).

Stability results showed that after 20 hours at room temperature, the RSD of curcumin was 0.63%, indicating that the CUR samples basically remained stable after 20 h at room temperature(Table S3).

Table S1. Intraday precision of CUR solution in release studies (*n* = 3)

C(μg·mL^{-1})	0 h	2 h	4 h	6 h	8 h	RSD（%）
5	4.7769	4.8094	4.8516	4.8546	4.8965	0.91
50	50.4536	50.6435	50.8264	51.0665	51.1663	0.58
100	99.9934	100.3530	100.9740	101.0224	101.2840	0.53

Table S2. Interday precision of CUR solution in release studies (*n* = 3)

C(μg·mL^{-1})	1 d	2 d	3 d	4 d	5 d	RSD（%）
5	4.8965	4.8990	4.965	5.0429	5.0923	1.68
50	51.1664	51.0153	52.1993	52.0444	52.381	1.20
100	50.4537	50.8264	51.1799	51.2372	51.0629	1.32

Table S3. Stability of CUR solution in release studies (*n* = 3)

浓度（μg·mL^{-1}）	0 h	4 h	8 h	12 h	20 h	RSD(%)
50	50.4537	50.8264	51.1799	51.2372	51.0629	0.63

1.5 Recovery Test

A fixed amount of C60-CPP5/Pser was weighed and added into CUR (feeding ratio 40 %:100 %:120 %) .The mixture was ultrasonically dispersed, diluted to 25 mL, and filtered through a 0.45 μm membrane filter. A 10 μL aliquot was injected into the HPLC system to determine the concentration of CUR.The recovery rates were 101.76%, 101.46%, and 100.59%, with RSD values of 1.20%, 1.31%, and 1.56%, respectively (Table S4). These results indicate that the spik recovery was acceptable and met the requirements of the assay.

Table S4.Spike recovery of curcumin in release studies (n = 3)

Material ratio (%)	$\bar{X}\pm SD$	RSD (%)
40	101.76 % ± 1.22 %	1.20
100	101.46 % ± 1.33 %	1.31
120	100.59 % ± 1.58 %	1.56

2. Method Validation for Intestinal Mucus Penetration Study

2.1 Chromatographic Conditions

Column: Hypersil GOLD™ C18 (200 mm × 4.6 mm, 5 μm);

Mobile phase: acetonitrile-0.1% phosphoric acid aqueous solution (48:52, v/v);

Flow rate: 1 mL·min^{-1};

Column temperature: 25 °C;

Detection wavelength: 430 nm;

Injection volume: 10 μL.

2.2 Specificity

Blank intestinal mucus solution, CUR standard solution, and CUR intestinal mucus solution were prepared. A 1 mL sample of each was transferred into a 1.5 mL EP tube and centrifuged at 12,000 rpm^{-1} for 10 minutes at low temperature,take the supernatant, and repeat two times.The final supernatant was analyzed by HPLC to determine the concentration of CUR in the mucus samples.

As shown in Figure S3, the curcumin peaks in the CUR intestinal mucus samples were well shaped, and the components in the mucus and the blank PBS solution did not interfere with the determination of CUR.

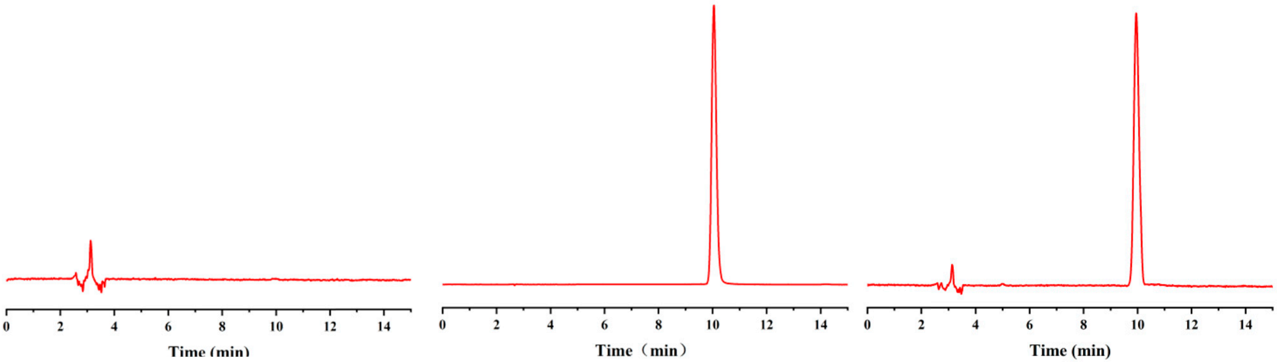

A Blank intestinal mucus solution B CUR standard solution C CUR intestinal mucus solution

Figure S3. Specificity of CUR mucus permeable samples.

2.3 Standard Curve

An appropriate amount of CUR reference substance was accurately weighed and dissolved to prepare a mother liquor of 100 μg·mL^{-1} using a solvent mixture of 50% ethanol and 50% PBS. The stock solution was serially diluted and analyzed by HPLC.A standard calibration curve was constructed by plotting the peak area of curcumin (*A, mAU*) on the y-axis against its concentration (*C, μg·mL^{-1}*) on the x-axis, using linear least-squares regression.

Curcumin in intestinal mucus samples exhibited good linearity over the concentration range of 1~20 μg·mL^{-1}. The regression equation was:$y = 0.5981x - 0.0933$(R^2 = 0.9998).The standard curve is shown in Figure S4.

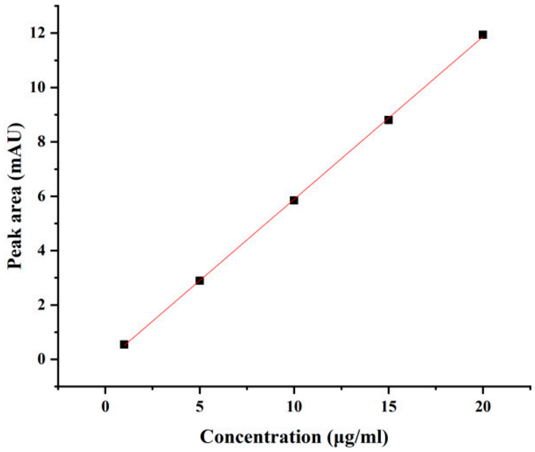

Figure S4. Standard calibration curve of CUR in mucus samples.

2.4 Precision and Stability

To evaluate intraday and interday precision, a CUR standard solution (100 μg·mL^{-1}) was diluted to three concentration levels: low (1 μg·mL^{-1}), medium (10 μg·mL^{-1}), and high (20 μg·mL^{-1}). Each concentration was sampled at 2-hour intervals, and CUR content was measured by HPLC in triplicate to assess intraday precision. The same samples were analyzed once daily for five consecutive days to assess interday precision.

To evaluate sample stability, a 10 μg·mL^{-1} CUR standard solution was stored at room temperature. Samples were collected at 0, 4, 8, 12, and 20 hours and analyzed by HPLC to determine CUR concentration.

As shown in Tables 5 and 6, the intraday RSD values were 0.17%, 0.32%, and 0.16% for low, medium, and high concentrations, respectively. The interday RSD values were 1.97%, 2.00%, and 1.94%, indicating good method precision. Stability results (Table S7) showed that the curcumin mucus samples remained stable at room temperature for up to 20 hours.

Table S5. Intraday precision of CUR solution for Intestinal Mucus Penetration Study (n = 3)

C(μg·mL^{-1})	0 h	2 h	4 h	6 h	8 h	RSD(%)
1	1.0729	1.0913	1.0783	1.1058	1.1058	0.17
10	9.9351	9.9126	9.8987	9.8564	9.8743	0.32
20	19.7900	19.7394	19.7405	19.7062	19.7196	0.16

Table S6. Interday precision of CUR solution for Intestinal Mucus Penetration Study (n = 3)

C(μg·mL^{-1})	1 d	2 d	3 d	4 d	5 d	RSD(%)
1	1.0729	1.0573	1.0991	1.0547	1.0706	1.97
10	9.9349	9.5084	9.4264	9.5551	9.5496	2.0
20	19.7900	18.9059	18.9919	18.946	19.0599	1.94

Table S7. Stability of CUR solution for Intestinal Mucus Penetration Study (n = 3)

C(μg·mL^{-1})	0 h	4 h	8 h	12 h	20 h	RSD(%)
10	9.9351	9.8987	9.8743	9.8839	9.8676	0.28

2.5 Recovery Test

A fixed amount of C60-CPP5/Pser was weighed and added into CUR (feeding ratio 40 %∶100 %∶120 %) .The mixture was diluted to 25 mL with a 50% anhydrous ethanol and 50% PBS solution, ultrasonically dispersed, and filtered through a membrane filter. The filtrate was analyzed by HPLC, and recovery was calculated.

The recovery rates were 101.35%, 97.74%, and 98.51%, with RSD values of 1.04%, 1.52%, and 1.09%, respectively. The results met the requirements for quantitative analysis (Table S8).

Table S8. Recovery of curcumin in mucus matrix for Intestinal Mucus Penetration Study (n = 3)

Material ratio(%)	$\bar{X} \pm SD$	RSD(%)
40	101.35% ± 1.06%	1.04
100	97.74% ± 1.49%	1.52
120	98.51% ± 1.14%	1.09

3. Method Validation for Cellular Uptake Assay

3.1 Chromatographic Conditions

Column: Hypersil GOLD™ C18 (200 mm × 4.6 mm, 5 μm);

Mobile phase: acetonitrile-0.1% phosphoric acid aqueous solution (48:52, v/v);

Flow rate: 1 mL·min^{-1};

Column temperature: 25 °C;

Detection wavelength: 430 nm;

Injection volume: 10 μL.

3.2 Sample Preparation for Cellular Uptake

A 200 μL aliquot of the cell sample was transferred to a 1.5 mL EP tube and centrifuged at 12,000 rpm^{-1} for 10 minutes at low temperature and and repeat two times.The final supernatant was analyzed by HPLC to determine the concentration of curcumin (CUR) in the sample.

3.3 Standard Curve

A total of 10 mg of CUR reference standard was accurately weighed and dissolved in 1 mL of DMSO. The resulting solution was diluted with PBS (pH 7.4) to prepare a 1 mg/mL mother liquor. This solution was serially diluted, and 10 μL was injected into the HPLC system.A standard calibration curve was constructed by plotting the peak area (*A, mAU*) on the y-axis against the concentration (*C, μg·mL*$^{-1}$) on the x-axis using linear least-squares regression.

Curcumin exhibited good linearity in the range of 1~100 μg·mL^{-1}. The regression equation was:y = 0.972x + 0.022 (R^2 = 0.9995).The standard curve is shown in Figure S5.

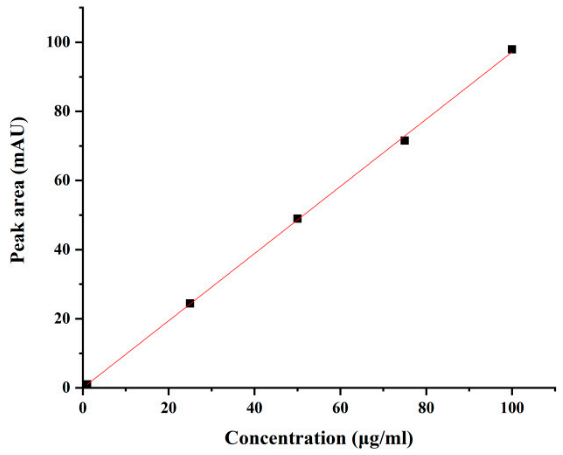

Figure S5. Standard calibration curve of CUR in cellular uptake samples.

4. Method Validation for Single-Pass Intestinal Perfusion Study

4.1 Sample Preparation

Sodium chloride (7.8 g), potassium chloride (0.35 g), sodium bicarbonate (1.37 g), sodium dihydrogen phosphate (0.32 g), and magnesium chloride (0.02 g) were dissolved in an appropriate volume of distilled water. Separately, calcium chloride (0.37 g) and glucose (1.4 g) were each dissolved in water with continuous stirring. All solutions were then combined and diluted with distilled water to a final volume of 1000 mL to obtain Krebs-Ringer buffer (Krebs solution) with pH=7.4.

Take 0.5 mL of the intestinal circulation sample in a 1.5 mL Ep tube, centrifuge at 3000 r·min^{-1} for 10 min, take the supernatant, add methanol to precipitate the protein,

vortex mixing, centrifuge at 12000 r·min^{-1} for 5 min, and then feed into the sample for determination by HPLC.

4.2 Specificity

Blank Krebs-Ringer buffer, phenol red intestinal perfusate (a mixture of phenol red, CUR, and blank intestinal perfusate), CUR standard solution, and CUR-phenol red intestinal perfusate were processed following the method described in section 4.1 and analyzed by HPLC.

The high performance liquid chromatography of curcumin enterocyclic samples is shown in Fig. 6, Krebs solution, phenol red, and intestinal perfusate did not interfere with the determination of the drug.

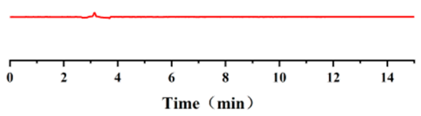

A Blank Krebs solution

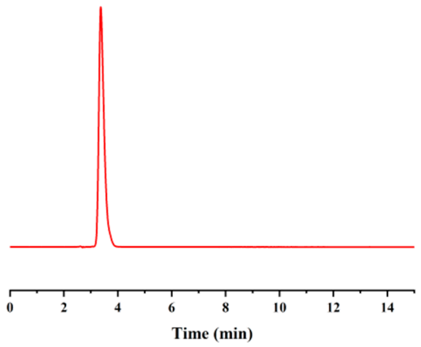

B Phenol red intestinal perfusate

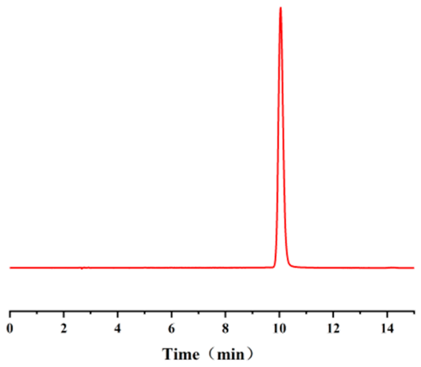

C CUR standard solution

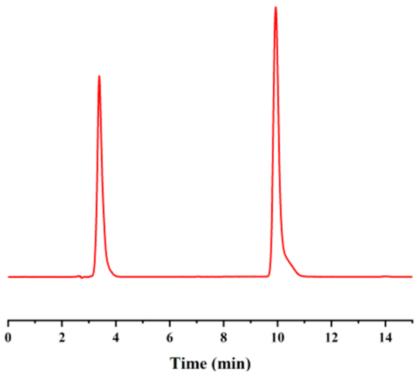

D CUR-phenol red intestinal perfusate

Figure S6. HPLC demonstrating specificity of CUR in single-pass intestinal perfusion samples.

4.3 Standard Curve

CUR stock solution and phenol red stock solution were mixed and diluted with blank intestinal perfusate to prepare a series of solutions with concentrations of 5, 10, 20, 30,

40, and 50 μg·mL^{-1}. These samples were processed according to the procedure described in section 4.1, and 10 μL of each was injected into the HPLC system.

A standard calibration curve was generated by plotting the peak area of CUR (*A, mAU*) on the y-axis against the concentration (*C, μg·mL^{-1}*) on the x-axis using linear least-squares regression.

Curcumin showed good linearity in the range of 5~50 μg·mL^{-1}. The regression equation was:$y = 0.6466x - 0.0226$ ($R^2 = 0.9996$).The standard curve is shown in Figure S7.

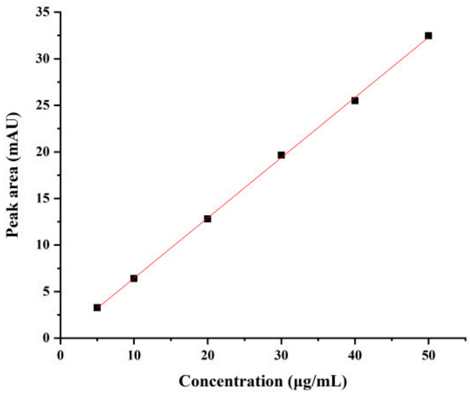

Figure S7. Standard calibration curve of CUR in intestinal perfusate samples.

4.4 Intraday and Interday Precision, and Stability

CUR stock solution and phenol red stock solution were mixed and diluted with blank intestinal perfusate to prepare samples at concentrations of 10, 30, and 50 μg·mL^{-1}. To assess intraday precision, samples at each concentration were collected three times within one day at 2-hour intervals. They were processed according to the method described in section 4.1 and analyzed by HPLC to calculate RSD values.For interday precision, the same concentrations were sampled three times per day over five consecutive days and processed in the same way for HPLC analysis.

To evaluate sample stability, a 30 μg·mL^{-1} CUR sample solution was stored at room temperature. Aliquots were collected at 0, 2, 4, 8, 12, and 24 hours, processed as described in section 4.1, and analyzed by HPLC.

As shown in Tables 9 and 10, the intraday RSDs were 0.08%, 0.06%, and 0.14%, while the interday RSDs were 1.01%, 1.05%, and 1.08% for the three concentrations, respectively, indicating good method precision. Stability results (Table S11) showed that the 30 $\mu g \cdot mL^{-1}$ curcumin intestinal perfusate sample remained stable at room temperature for up to 24 hours.

Table S9. Intraday precision of CUR in intestinal perfusate (*n* = 3)

C($\mu g \cdot mL^{-1}$)	0 h	2 h	4 h	8 h	12 h	RSD(%)
10	9.8569	9.8701	9.8656	9.8795	9.8649	0.08
30	29.7456	29.7113	29.7134	29.7282	29.7051	0.06
50	49.8604	49.7826	49.7213	49.7346	49.7346	0.14

Table S10. Interday precision of CUR in intestinal perfusate (*n* = 3)

C($\mu g \cdot mL^{-1}$)	1 d	2 d	3 d	4 d	5 d	RSD(%)
10	9.8569	10.0114	9.8248	9.7713	9.7719	1.01
30	29.7444	29.9799	29.3949	29.2657	29.3118	1.05
50	49.8604	49.7693	49.0162	48.6992	48.9038	1.08

Table S11. Stability of CUR in intestinal perfusate at room temperature (*n* = 3)

C($\mu g \cdot mL^{-1}$)	0 h	2 h	4 h	8 h	12 h	24h	RSD(%)
30	29.7456	29.7113	29.7134	29.7283	29.7051	29.7444	1.08

4.5 Recovery Test

A fixed amount of C60-CPP5/Pser was weighed and added into CUR (feeding ratio 10 %：30%：100 %) .After mixing with phenol red stock solution, the mixtures were diluted with blank intestinal perfusate to 10 mL. The samples were processed according to the method described in section 4.1 and analyzed by HPLC to calculate the recovery.

The results showed that the recovery rates were (101.32 ± 0.18)%, (99.19 ± 0.37)%, and (100.45 ± 0.17)% for the three spiking levels. The RSD values were all below 2%, indicating that the recovery was acceptable and met analytical requirements.

4.6 Standard Curve of Phenol Red

Phenol red was serially diluted with Krebs-Ringer buffer. A 0.5 mL aliquot of each dilution was mixed with 1 mmol·mL^{-1} NaOH solution to develop color, and the absorbance was measured at 559 nm. A standard calibration curve was constructed by plotting phenol red peak area(*A, mAU*) on the y-axis， curcumin concentration (*C*， *μg·mL^{-1}*) on the x-axis using linear least-squares regression.

Phenol red in Krebs-Ringer buffer showed good linearity in the range of 1~100 μg·mL^{-1}. The regression equation was:$y = 0.4283x - 0.1117$ ($n = 3$, $R^2 = 0.9998$).The standard curve is shown in Figure S8.

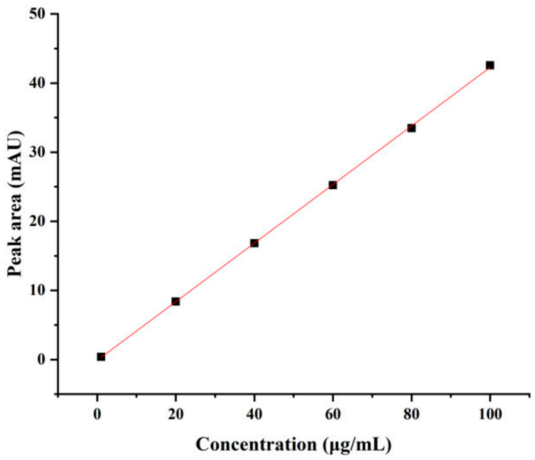

Figure S8. Standard calibration curve of phenol red in Krebs-Ringer buffer.

5. Method Validation for Plasma Sample Processing in Pharmacokinetic Study

5.1 Mass Spectrometry Conditions

Column: Agilent ZORBAX SB-C18 (150 mm × 4.6 mm, 5 μm);

Mobile phase: acetonitrile–formic acid aqueous solution (48:52, v/v);

Flow rate: 1 mL·min^{-1};

Column temperature: 30 °C;

Ionization source: electrospray ionization (ESI);

Scan mode: positive ion;

Acquisition mode: multiple reaction monitoring (MRM);

Capillary voltage: 3.0 kV;

Collision gas: nitrogen.

5.2 Plasma Sample Preparation

A 100 μL aliquot of plasma sample was mixed with 20 μL of resveratrol internal standard solution (100 μg·mL^{-1}). The mixture was extracted twice with 500 μL of ethyl acetate each time, vortexed for 3 minutes, and centrifuged at 12000 r·min^{-1} for 10 minutes. The supernatants were combined and evaporated to dryness under vacuum at 40 °C. The residue was reconstituted in 100 μL of chromatographic-grade methanol, centrifuged again at 12000 r·min^{-1}, and the supernatant was analyzed by HPLC-MS.

5.3 Standard Curve

Blank plasma (200 μL) was spiked with serial dilutions of CUR stock solution to prepare calibration samples at different concentrations. A 10 μL aliquot of each was injected, and the peak areas were recorded. A standard calibration curve was constructed by plotting the ratio of CUR peak area to internal standard peak area (y-axis) against drug concentration (C, ng·mL^{-1}, x-axis), using linear least-squares regression.

Curcumin showed good linearity over the concentration range of 0.1~1000 ng·mL^{-1}. The regression equation was:$y = 0.0035x + 0.0267$ ($R^2 = 0.9955$).The standard curve is shown in Figure S9.

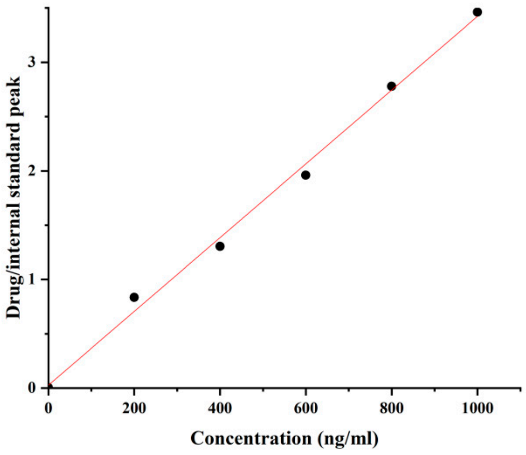

Figure S9. Standard calibration curve of CUR in plasma.

5.4 Intraday and Interday Precision, and Stability

Blank plasma was spiked with CUR stock solution to prepare plasma samples at three concentration levels: low (10 ng·mL^{-1}), medium (400 ng·mL^{-1}), and high (800 ng·mL^{-1}). To assess intraday precision, samples were collected three times within one day at 2-hour intervals, processed according to the method described in section 5.2, and analyzed by HPLC-MS. For interday precision, the same samples were collected three times per day over five consecutive days and analyzed using the same procedure.

To assess stability, 400 ng·mL^{-1} CUR plasma samples were prepared by mixing blank plasma with CUR stock solution. The samples were incubated at 37 °C, and aliquots were taken at 0, 2, 4, 8, and 24 hours. Each aliquot was processed as described in section 5.2 and analyzed by HPLC-MS.

As shown in Tables 12 and 13, the intraday RSDs for curcumin at concentrations of 10, 400, and 800 ng·mL^{-1} were 14.58%, 10.24%, and 4.14%, respectively. The interday RSDs were 17.63%, 8.04%, and 3.05%, respectively. These results indicate acceptable precision at higher concentrations.

Stability data (Table S14) showed that CUR remained relatively stable at room temperature over 24 hours.

Table S12. Intraday precision of CUR in plasma (*n* = 3)

C(ng·mL^{-1})	0 h	2 h	4 h	8 h	12 h	RSD(%)
10	8.3714	9.5143	6.3714	7.8000	7.5143	14.58
400	393.9429	396.9143	517.8857	455.4857	473.4857	10.24
800	780.0571	820.3714	786.0571	803.4000	726.6286	4.14

Table S13. Interday precision of CUR in plasma (*n* = 3)

C(ng·mL^{-1})	1 d	2 d	3 d	4 d	5 d	RSD(%)
10	8.3714	8.0857	11.5143	7.8000	8.0857	17.63
400	393.9429	473.4857	434.0857	425.6286	481.8571	8.04
800	786.0571	820.3714	773.9429	782.6286	754.0000	3.05

Table S14. Stability of CUR in plasma at room temperature (*n* = 3)

$C(ng\cdot mL^{-1})$	0 h	2 h	4 h	8 h	24 h	RSD(%)
400	393.9429	396.9143	517.8857	455.4857	473.4857	11.58
