# Supplementary material for: An Effective Oral Nanodelivery Material for Curcumin: Ingenious Utilization of Gastrointestinal Absorption Characteristics
Source: Molecules. 2025 Jun 10;30(12):2536. doi: 10.3390/molecules30122536 (PMC12196007; doi:10.3390/molecules30122536)
Supplement: Supplementary file 1 [file molecules-30-02536-s001.zip › supplymentary materials-S2.pdf]

Supplement Material-S2

The single-pass intestinal perfusion study in rats demonstrated that the carrier significantly enhanced CUR absorption across all intestinal segments, with absorption kinetics conforming to first-order models. Notably, duodenal and colonic absorption exhibited the most pronounced improvements. At 2 h, the cumulative absorption percentage of the carrier formulation was 1.28 times higher than CUR solution in the duodenum, followed by 1.12 times in the jejunum, 1.15 times in the ileum, and 1.69 times in the colon (Table S1).

Table S1. Percentage of residual dose of CUR in different groups.

**Intestinal segment**	**Time (min)**	**Remaining dosage (%)**	
		**Free CUR solution**	**C60-CPP5/Pser@CUR**
Duodenum	15	93.61 ± 0.59	91.80 ± 0.81
	30	89.22 ± 1.18	80.24 ± 2.41
	45	79.11 ± 0.58	72.54 ± 0.94
	60	69.18 ± 1.92	67.15 ± 0.92
	75	61.93 ± 1.54	58.62 ± 1.03
	90	54.95 ± 0.33	42.59 ± 1.19
	105	45.36 ± 1.72	30.17 ± 5.12
	120	42.13 ± 0.88	25.90 ± 0.57
Jejunum	15	94.43 ± 2.05	92.41 ± 0.91
	30	90.52 ± 0.80	86.24 ± 0.80
	45	87.97 ± 0.43	77.19 ± 0.85
	60	77.07 ± 0.97	68.79 ± 2.19
	75	70.44 ± 0.82	56.38 ± 1.03
	90	62.99 ± 0.92	49.46 ± 1.09
	105	54.99 ± 0.74	44.47 ± 1.08
	120	46.26 ± 0.94	39.43 ± 1.07
Ileum	15	93.68 ± 0.37	92.01 ± 0.92
	30	89.84 ± 0.89	83.35 ± 0.84
	45	82.04 ± 1.06	75.71 ± 0.55
	60	72.17 ± 1.08	66.60 ± 0.31
	75	64.11 ± 1.25	54.81 ± 0.70
	90	58.61 ± 0.21	46.83 ± 0.68
	105	51.48 ± 1.18	40.94 ± 0.96
	120	45.11 ± 3.27	36.44 ± 1.09
Colon	15	97.47 ± 0.83	93.23 ± 1.50
	30	92.6 ± 1.04	88.27 ± 2.52
	45	87.97 ± 1.07	81.23 ± 1.00
	60	79.51 ± 3.48	73.64 ± 0.79
	75	78.03 ± 0.99	68.20 ± 1.13
	90	73.23 ± 0.45	58.02 ± 1.12
	105	70.98 ± 0.98	50.26 ± 4.79
	120	66.55 ± 1.07	43.26 ± 0.75

The effective permeability coefficient (Peff) of C60-CPP5/Pser@CUR in the duodenum, jejunum, ileum, and colon demonstrated 2.08, 1.26, 1.27, and 2.37 times enhancements compared to free CUR solution, respectively (Table S2).

Table S2. Absorption constant and surface permeability coefficient of C60-CPP5/Pser@CUR in different intestinal segment.

Group	Ntestinal segment	Ka (min^{-1})	Peff×10^{-5} (cm/s)
CUR	Duodenum	0.52 ± 0.0048	2.72 ± 0.05
	Ileum	0.47 ± 0.0208	2.33 ± 0.11
	Jejunum	0.47 ± 0.0088	2.61 ± 0.37
	Colon	0.29 ± 0.0095	0.83 ± 0.01
C60-CPP5/Pser	Duodenum	0.64 ± 0.0249	5.66 ± 0.15
	Ileum	0.56 ± 0.0019	2.95 ± 0.14
	Jejunum	0.54 ± 0.0108	3.33 ± 0.03
	Colon	0.49 ± 0.0167	1.97 ± 0.06
